# Seasonal fluctuation of lung function in cystic fibrosis: A national register-based study in two northern European populations

**DOI:** 10.1016/j.jcf.2018.10.006

**Published:** 2019-05

**Authors:** Tavs Qvist, Daniela K. Schlüter, Vian Rajabzadeh, Peter J. Diggle, Tania Pressler, Siobhán B. Carr, David Taylor-Robinson

**Affiliations:** a Copenhagen Cystic Fibrosis Centre, Department of Infectious Diseases, Rigshospitalet, Copenhagen University, Copenhagen, Denmark; b Centre for Health Informatics, Computing and Statistics (CHICAS), Lancaster Medical School, Lancaster University, Lancaster LA1 4YW, United Kingdom; c Centre for Primary Care and Public Health, Queen Mary University of London, United Kingdom; d Department of Respiratory Paediatrics, Royal Brompton Hospital, London, United Kingdom; e Department of Public Health and Policy, Farr Institute, University of Liverpool, Liverpool L69 3GB, United Kingdom

**Keywords:** Seasonality, Season, Fluctuation, Percent predicted FEV1

## Abstract

**Background:**

Many risk factors for lung disease in cystic fibrosis (CF) display a seasonal pattern yet it is unclear whether this is reflected in seasonal fluctuations in lung function.

**Methods:**

We conducted a longitudinal study using CF registries in Denmark and the UK. 471 individuals with a median of 104 FEV_1_ measurements per person and 7586 individuals with a median of nine FEV_1_ measures per person were included from Denmark and the UK respectively. We estimated the effect of seasonality on percent predicted FEV_1_ trajectories using mixed effects models whilst adjusting for clinically important covariates.

**Results:**

We found no significant cyclical seasonal variation in lung function in either country. The maximum variation in percent predicted FEV_1_ around the yearly average was estimated to be 0.1 percentage points (95%CI 0 to 0.21) and 0.14 percentage points (95%CI 0 to 0.29) in Denmark and the UK, respectively. When considering possible step-like changes between the four seasons, we found that lung function was higher in spring compared to winter in the UK (0.34 percentage points, 95%CI 0.1 to 0.59) though the difference was not of clinical significance.

**Conclusion:**

In both the UK and Denmark there may be small seasonal changes in lung function but this effect is not of clinical importance.

## Highlights

• It is not well understood how the four seasons affect lung function in CF.
• We performed a longitudinal analysis in the UK and Danish CF populations.
• Forty years of data were included in a comprehensive mixed effects models.
• The seasonal variation was small and not clinically important.

## 1 Introduction

Seasonal patterns in respiratory outcomes in conditions such as asthma and COPD are well recognised in terms of lung function, admissions to hospital and deaths [1,2]. Furthermore, theories about seasonal variations in cystic fibrosis (CF) morbidity are common, though they often rely more on shared observations and clinical hunches than hard evidence. For example in the early days of CF medicine a puzzling seasonal trend in CF mutations at birth was observed [3], but quickly debunked as a case of ascertainment bias [4]. It is perhaps not surprising that theories on climate and seasonal variations are common in CF; weather is a popular topic, probably because it is a shared experience, ripe for pattern-finding [5,6]. More importantly, many of the significant risk factors associated with CF pulmonary disease display seasonal variability. The most well-known examples are seasonal influenza outbreaks, which lead to increased mortality in the general population [7] and in CF populations, where rates of pulmonary exacerbations increase during epidemics [8]. Likewise, acquisition of *Pseudomonas aeruginosa* has been shown to exhibit seasonal variation, such that US children with CF in temperate and continental climate zones have a higher incidence in summer months [9], with no difference among children in dry climate zones. Conversely, an early Danish study showed more *Pseudomonas* acquisition in winter [10].

In the context of a multitude of interacting risk and protective factors influencing CF outcomes over time, and given the inherent variability in lung function measures in people with CF [11], any seasonality effect on risk factors and clinical outcomes is difficult to isolate. Furthermore it is unclear whether seasonality affects lung function, the main clinical morbidity indicator in CF. We therefore developed a longitudinal model for evaluating lung function changes over time in people with CF, and applied it to two CF populations in Northern Europe. The aim of our study was to assess seasonal fluctuations in lung function at the population level.

## 2 Methods

### 2.1 Study design, setting, data sources and participants

We carried out longitudinal analyses of lung function in individuals with CF captured in the Danish and UK CF registers between 1974 and 2014 and between 1996 and 2015, respectively. Individuals born before 1969 were excluded to reduce the influence of survivor bias [11]. Lung function measurements taken post-transplant or before the age of five were also excluded.

In Denmark, individuals with CF were followed up monthly in one of the two CF centres in Copenhagen and Aarhus. Measurements were recorded in the Danish CF Patient Registry, which was established in 1974 but includes records going back to the 1960s and has an almost complete record of all individuals living with CF in Denmark from 1990 onwards when CF care was centralised. In the UK individuals with CF were seen in one of 50 specialist CF centres. The recommendation in the UK is that the annual encounter data submitted to the Registry is from a clinic visit roughly 12 months after the previous entry and when the patient is clinically stable. Records date back to the 1990s and are estimated to capture 99% of the current UK CF population (see [12] for more details). In both countries, the follow-up reviews included evaluation of clinical status, lung function, and microbiology of respiratory tract secretions.

### 2.2 Outcome, exposure and covariates

Our outcome of interest was lung function from age 5 as measured by percent of predicted forced expiratory volume in one second (%FEV_1_). Pulmonary function tests were performed at the monthly/annual review visits. Measurements were expressed as a percentage of predicted values for sex and height in Denmark [13,14], and as a percentage of predicted values for sex, age, height and ethnicity in the UK [15]. Our exposure of interest was the time of year of measurement. Two variables were created for this. One was a 4 level categorical variable for the season during which the review visit took place, where December, January, and February were coded as ‘winter’, March, April, and May as ‘spring’, June, July and August as ‘summer’ and September, October, November as ‘autumn’. Our other approach was to use the day of year of the review visit, where 1st January was day zero and 31st December was day 365/366.

In both populations we adjusted for the following time-invariant covariates: age at diagnosis, birth cohort, pancreatic insufficiency (PI, coded as 0 or 1 according to whether PI was ever diagnosed), genotype (coded as the number of F508del alleles (0, 1 or 2)) and sex. We also adjusted for age and CF related diabetes (CFRD) as well as chronic pseudomonas as time varying covariates (the latter two coded as 0 or 1). In the UK we additionally included deprivation z-score based on the index of multiple deprivation (IMD), ethnicity (grouped as White, Black, North East Asian, South East Asian, Other/Mixed) and a binary indicator for diagnosis by new-born screening.

### 2.3 Statistical analysis

We developed a longitudinal model for lung function in Denmark and in the UK using a previously published approach [11,16]. In brief, we developed a linear model for the population average lung function, in which both the intercept at age 5 and the slope depend on the time-invariant covariates and the slope additionally depends on CFRD and chronic pseudomonas. The lung function measurements within an individual are correlated, but as the healthcare systems differ between countries, we cannot assume the underlying stochastic process to be the same in the Danish and UK CF populations and we therefore modelled the data from each country separately. Due to the different follow-up procedure, the short term-correlation that is captured in Denmark cannot be quantified in the UK. Therefore, we used different models for the longitudinally structured correlation; in Denmark we used an exponentially decaying function of time difference [11] whereas in the UK we used a random slope model [16]. Both models included a random intercept to take into account the between individual heterogeneity in baseline lung function. See Supplementary Material for the model equation and further details.

To assess whether there were seasonal fluctuations in lung function, we added the time of year as a time-varying covariate to the model using two different approaches. In the first approach we used a categorical variable with the levels ‘winter’, ‘spring’, ‘summer’ and ‘autumn’ as an explanatory variable of lung function. We used ‘winter’ as the reference level as it may be plausible that lung function is lowest during this time. In the second approach, we modelled smooth changes in lung function according to season using a sine wave where the period is one year (365.25 days) and the amplitude and horizontal shift are model parameters to be estimated from the data (see Supplementary Material for further details).

We fitted the model using maximum likelihood estimation (ML) and the R package nlme [17]. Statistical significance of a seasonal effect was assessed with a likelihood ratio test. Confidence intervals for the categorical ‘season’ variable were constructed using the R function intervals.lme, which uses a normal approximation of the ML estimators for the average differences in lung function in spring, summer and autumn compared to winter. For the approach using the sine wave, confidence intervals for the amplitude and the phase shift were also based on a normal approximation (more details are given in the Supplementary Material). Only individuals with complete information on the baseline covariates were included in the analysis.

### 2.4 Robustness test and additional analysis

As a robustness test, we repeated the analysis dropping measurements taken from individuals born before 1991 in the UK to reduce any remaining potential influence of survivor bias in this population. To assess whether the seasonal patterns differed between children and adults we re-fitted the models with the sine wave in both countries including an interaction term between the sine function and an indicator for < or ≥ 18 years of age.

### 2.5 Ethical considerations

NHS research ethics approval (Huntingdon Research Ethics Committee 07/Q0104/2) was granted for the collection of data into the UK database. The Cystic Fibrosis Trust database committee approved the use of anonymised data in this study. In Denmark the study was approved by the Danish Data Protection Agency (file no. 2008-41-2682).

### 2.6 Role of the funding source

This work was funded by the UK Cystic Fibrosis Trust through the Strategic Research Centre “CF EpiNet: Harnessing data to improve lives”. DTR was also funded by the MRC on a Clinician Scientist Fellowship (MR/P008577/1). The funder was not involved in the study design, data collection, data analysis, data interpretation, or in the writing of the report. The corresponding author had full access to all the data in the study and final responsibility for the decision to submit for publication.

## 3 Results

### 3.1 Participants

485 individuals in the Danish CF Registry were born between 1969 and 2009 all of whom had at least one lung function measurement after the age of 5. Age at diagnosis was missing in 14 individuals who were thus excluded from the analysis. The median follow-up time was 12.6 years with a median of 104 FEV_1_ measures per patient. In the UK CF Registry 10,269 individuals were born between 1969 and 2010; 9667 had lung function measurements after the age of 5 out of which 7586 had complete covariate data (see Supplementary Material for a comparison of the demographics). The median follow-up time in the UK study population was 10.4 years with a median of nine FEV_1_ measures. Table 1 gives the demographics of the study population stratified by birth cohort. Follow-up visits were approximately evenly distributed across the year in Denmark and increased slightly in frequency towards the end of the year in the UK (see Table 2).

**Table 1:** Demographics of the study population by country and birth cohort.

	Denmark						UK					
	1969–1977	1978–1987	1988–1997	1998–2007	2008–2009	Total	1969–1977	1978–1987	1988–1997	1998–2007	2008–2010	Total
n	97	110	144	113	7	471	790	1852	2546	2011	387	7586
Sex = male (%)	51 (52.6)	57 (51.8)	66 (45.8)	52 (46)	4 (57.1)	230 (48.8)	452 (57.2)	1023 (55.2)	1309 (51.4)	1003 (49.9)	190 (49.1)	3977 (52.4)
Ethnicity = white (%)	NA	NA	NA	NA	NA	NA	779 (98.6)	1799 (97.1)	2474 (97.2)	1906 (94.8)	362 (93.5)	7320 (96.5)
#F508del alleles(%)												
0	0 (0.0)	2 (1.8)	0 (0.0)	1 (0.9)	0 (0.0)	3 (0.6)	71 (9.0)	146 (7.9)	202 (7.9)	175 (8.7)	43 (11.1)	637 (8.4)
1	6 (6.2)	10 (9.1)	8 (5.6)	5 (4.4)	0 (0.0)	29 (6.2)	354 (44.8)	664 (35.9)	929 (36.5)	706 (35.1)	143 (37.0)	2796 (36.9)
2	91 (93.8)	98 (89.1)	136 (94.4)	107 (94.7)	7 (100.0)	439 (93.2)	365 (46.2)	1042 (56.3)	1415 (55.6)	1130 (56.2)	201 (51.9)	4153 (54.7)
Diagnosis by NBS (%)	NA	NA	NA	NA	NA	NA	12 (1.5)	92 (5.0)	257 (10.1)	444 (22.1)	286 (73.9)	1091 (14.4)
Mean IMD z-score (sd)	NA	NA	NA	NA	NA	NA	−0.10 (0.93)	0.02 (0.96)	0.02 (1.00)	−0.02 (1.01)	−0.07 (1.01)	−0.01 (0.99)
Pancreatic insufficient (%)	91 (93.8)	101 (91.8)	137 (95.1)	108 (95.6)	7 (100.0)	444 (94.3)	658 (83.3)	1690 (91.3)	2361 (92.7)	1813 (90.2)	323 (83.5)	6845 (90.2)
CFRD during study period (%)	37 (38.1)	33 (30.0)	27 (18.8)	1 (0.9)	0 (0.0)	98 (20.8)	404 (51.1)	882 (47.6)	931 (36.6)	271 (13.5)	6 (1.6)	2494 (32.9)
Chronic PA during study period (%)	71 (73.2)	52 (47.3)	37 (25.7)	10 (8.8)	0 (0.0)	170 (36.1)	632 (80.0)	1558 (84.1)	1792 (70.4)	577 (28.7)	27 (7.0)	4586 (60.5)
Mean age at diagnosis (sd)	4.15 (7.43)	2.42 (5.47)	1.95 (3.04)	1.84 (2.93)	0.21 (0.21)	2.46 (4.89)	9.08 (13.11)	3.69 (7.12)	2.08 (4.03)	1.11 (2.19)	0.19 (0.60)	2.85 (6.53)

NBS: Newborn bloodspot screening; IMD: Index of multiple deprivation where higher scores equate to higher deprivation; CFRD: CF related diabetes; PA: Pseudomonas Aeruginosa

**Table 2:** Number of FEV_1_ measurements (%) taken in the four season and their unadjusted mean values across all individuals (standard deviation).

Country	Winter		Spring		Summer		Autumn	
	Number of measurements	Mean value (% predicted)	Number of measurements	Mean value (% predicted)	Number of measurements	Mean value (% predicted)	Number of measurements	Mean value (% predicted)
Denmark	16,104 (25%)	74.97 (25.18)	16,505 (25%)	74.87 (25.2)	16,014 (24%)	74.87 (25.35)	16,851(26%)	74.95 (25.21)
UK	14,762 (21%)	71.1 (23.06)	16,084 (23%)	71.03 (23.04)	17,565 (25%)	71.11 (23.0)	22,256 (31%)	71.17 (23.17)

### 3.2 Seasonal effects on lung function

**Fig. 1:**
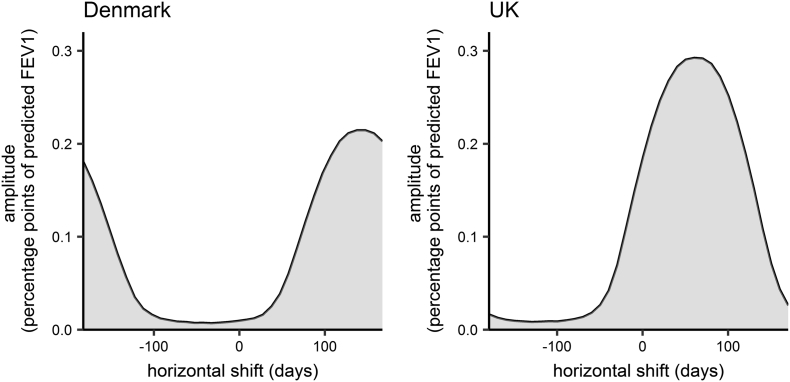
Joint 95% confidence region of the horizontal shift and amplitude in Denmark and the UK.

In the Danish population the overall effect of seasonality on lung function was not significant at the 5% level in either the model with the categorical season variable or the model with the sine function. Parameter estimates, confidence intervals and likelihood-ratio test *p*-values are given in Table 3. Lung function was not found to differ significantly between spring, summer or autumn and winter. Using the sine wave to capture smoothly varying seasonal fluctuations, we estimated an amplitude of 0.1 percentage points (95%CI 0 to 0.21). The horizontal shift was estimated to be 148.47 days (95%CI -182.32 to 182.61). Thus lung function was estimated to peak on the 28th August and dip on the 27th February. Confidence intervals for both dates covered the entire year. Amplitude and horizontal shift are however correlated; Fig. 1 shows their joint 95% confidence region. Only for a horizontal shift >63 days or less than −130 days, which equates to lung function peaking between June and October, was the upper 95% bound for the amplitude >0.05 (see Supplementary Material for details).

**Table 3:** Parameter estimates (95% confidence intervals) for the seasonal effects on percent predicted FEV_1_ and *p*-values from the Likelihood ratio test.

Parameters		Denmark		UK	
		Estimates	p-value	Estimates	p-value
Categorical ‘season’ variable (percentage points; reference level = winter)	Spring	0.01 (−0.15 to 0.18)	0.34	0.34 (0.1 to 0.58)	0.06
	Summer	0.1 (−0.07 to 0.28)		0.21 (−0.04 to 0.45)	
	Autumn	0.13 (−0.03 to 0.2)		0.17 (−0.06 to 0.39)	
Amplitude of sine wave (percentage points)		0.1 (0 to 0.21)	0.07	0.14 (0 to 0.29)	0.07
Horizontal shift of sine wave (days)		148.47 (−182.32 to 182.61)		66.2 (−182.25 to 179.51)

In the UK, the overall effect of seasonality on lung function was only marginally not significant at the 5% level. Lung function was estimated to be significantly higher in spring compared to winter (0.34 percentage points (95%CI 0.1 to 0.59)), whereas there was no significant difference between lung function in summer or autumn and winter. Using the sine function, we estimated an amplitude of 0.14 percentage points (95%CI 0 to 0.29). The horizontal shift was estimated to be 66.2 days (95%CI -182.25 to 179.51). Thus lung function was estimated to peak on the 7th June and dip on the 6th December, with confidence intervals for both dates covering the entire year. However, the upper 95% confidence limit for the amplitude was >0.05 only for horizontal shifts between −26 and 157 days, which equates to lung function peaking between March and September (see Fig. 1).

**Fig. 2:**
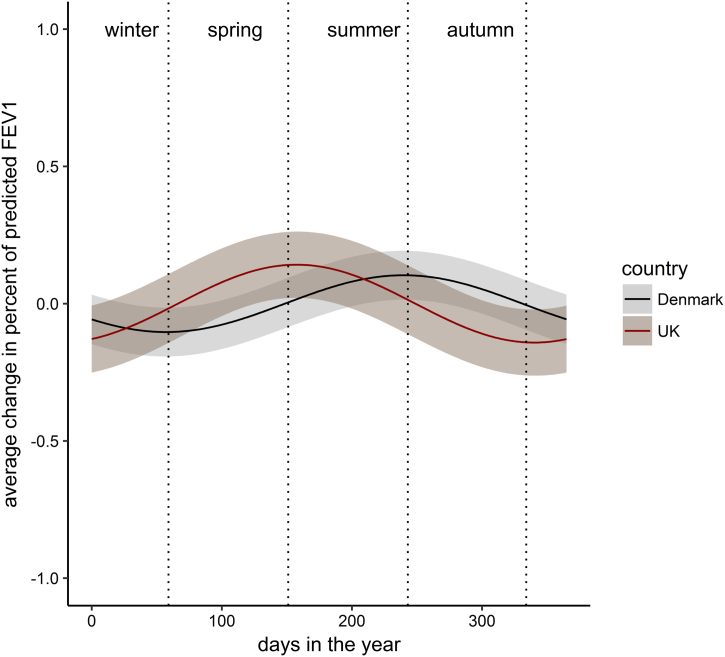
Estimated seasonal fluctuation in percent predicted FEV_1_. The shaded regions are the 95% confidence regions. 0 on the x-axis represents the 1st January.

Fig. 2 shows the modelled cyclical seasonal fluctuation in percent predicted FEV_1_ in both countries.

Tables S2 and S3 in the Supplementary Material give all parameter estimates and 95% confidence intervals for the covariates included in the model; Tables S4 and S5 in the Supplementary Material give the estimated Variance-Covariance parameters.

### 3.3 Robustness test and additional analysis

Repeating the analysis in the UK only on individuals with CF born after 1991 reduced the point estimates, but the confidence intervals were compatible with the previously presented results. Details are given in the Supplementary Material.

In the additional analysis, which included an interaction effect between the sine function and an indicator for < or ≥ 18 years of age, we did not find any significant differences in seasonal patterns in lung function between children and adults. See Supplementary Material for details.

## 4 Discussion

We carried out a longitudinal analysis of lung function change over time in two national Northern European CF populations and found that there is no clinically important seasonal variation in lung function.

Given the reported seasonal fluctuation of some risk factors for adverse CF outcomes, such as influenza epidemics and PA acquisition, the lack of any substantive seasonal variation in lung function is perhaps an unexpected finding. Rates of pulmonary exacerbations have been reported as being more frequent during influenza epidemics [8]. Acquisition of *Pseudomonas aeruginosa* has also been shown to demonstrate seasonal variation, either with a higher incidence in summer [9], or in winter [10], depending on the geographical location.

A clear seasonal variability in non-influenza respiratory viruses has also been observed in CF, but this season effect does not translate to pulmonary exacerbations [18]. A study from the CF Foundation National Patient Registry showed that MRSA was more frequently acquired in autumn and winter, whereas *A. xylosoxidans* acquisition was lower in spring. For *H. influenzae*, winter and spring were associated with higher acquisition. No seasonal variation was observed for *S. maltophilia* acquisition [19]. It is well established that there are higher mould counts in the outdoor environment in autumn and this has been suspected to lead to higher risks of pulmonary *Aspergillus* and ABPA [20], but other reports have found that even adequate climate conditions for *Aspergillus* are in themselves not ideal conditions for increased acquisition [21]. Other environmental factors that show seasonal variability, but where influence on morbidity in CF is unclear, include ambient temperatures [22], air humidity and domestic water temperature [23]. All of these might be expected to affect the prevalence or virulence of well-known CF pathogens, but clear evidence is lacking. The connection between season, pathogen acquisition and lung function in CF is thus clearly not straightforward.

In addition vitamin-D levels are also known to fluctuate with sun exposure during the calendar year. Such fluctuations might be expected to be mirrored in a hard outcome such as lung function, but this link has also turned out not to be direct [24]. Dehydration during warm weeks is a risk factor for obstipation, but not for lung function [25]. Altered clinic opening hours during vacation periods, patient travel patterns during the calendar year, and respiratory outbreaks during seasonal CF community events [26,27] could all effect groups of patients, but the effect on a population level is uncertain.

There are a number of potential explanations for the lack of seasonal variation in lung function in our study. Firstly, the size of any seasonal fluctuation in risk factors and subsequent impact on lung function may have previously been overestimated, or other, differently distributed and more dominant effects may lessen their impact. Secondly, it is possible that CF maintenance therapy and exacerbation management in the UK and Denmark is able to mitigate any negative effects of the winter season. A recent study in the US found that patients in the CFF Registry had a higher lung function in January compared to July. Similar to our findings, the difference at the population level was small and clinically insignificant at an average of about 1.2%FEV_1_ [28]. We found lung function to be higher in spring than in winter in the UK but did not find a statistically significant difference between winter and summer. The US study also showed that the effect of annual average temperatures on lung function dominated over seasonal fluctuations. The differences in climate between northern Europe and the US may therefore go some way in explaining any differences in findings.

### 4.1 Strengths and weaknesses

A strength of this analysis is that we analysed two well-characterised population-level CF registry datasets, with consistent findings across the two analyses, using up to date statistical approaches appropriate to the differing data collection pattern in the two datasets. The Danish dataset had monthly clinic visit frequency facilitating precise estimation of change within individuals over time. By contrast, the UK dataset contained many more individuals but had less frequent follow-up throughout the year, allowing more precise estimation of the cross-sectional effect of seasonality. A limitation of our analysis is that we did not have data on the precise date of onset of PA or other respiratory pathogens in either dataset, and thus were not able to assess if there was seasonal variation in these risk factors. Similarly, we did not have data on potential changes in CF management throughout the year, making it impossible to determine whether responses from clinical staff mitigate any potentially negative effects of seasonal changes in pathogens and environmental factors on lung function.

## 5 Conclusion

Our findings from the analysis of national CF registry populations in Denmark and the UK suggest that there is no clinically significant seasonality effect on lung function.

## Contributors

SBC, VR, TQ and DKS conceived the original idea for this study. DKS, TQ, SBC, PJD and DTR designed the study. DKS, PJD and DTR developed the analysis plan. DKS carried out the analysis. TQ, SBC and TP helped identify previous work and gave the clinical interpretation. TQ, DKS and DTR wrote the first draft of the paper. All authors were involved in interpreting the findings and revising drafts and agreeing the final version.

## Appendix A Supplementary data

Supplementary material Image 1
