# Supplementary material for: Seasonal fluctuation of lung function in cystic fibrosis: A national register-based study in two northern European populations
Source: J Cyst Fibros. 2019 May;18(3):390–5. doi: 10.1016/j.jcf.2018.10.006 (PMC6559396; doi:10.1016/j.jcf.2018.10.006)
Supplement: Supplementary file 1 — Supplementary material [file mmc1.pdf]

Supplementary Material

The effect of season on lung function in cystic fibrosis: A national register based study in two northern European populations

Methods

Model for longitudinal lung function trajectories without seasonal effects

Let y_{ij} denote the jth observed value for % predicted FEV_1 in individual i. We model the longitudinal FEV_1 trajectories by

$$y_{ij} = x_i'\beta_1 + x_i't_{ij}\beta_2 + CFRD_i(t_{ij})(t_{ij} - t_{ik_i})\beta_3 + cPA_i(t_{ij})(t_{ij} - t_{il_i})\beta_4 + V_i + W_i(t_{ij}) + Z_{ij}$$

where x_i is the vector of time-invariant covariates values for individual i (age at diagnosis, birth cohort, sex, genotype, pancreatic insufficiency; in the UK additionally IMD and ethnicity),

t_{ij} is time of observation j in individual i measured as time since age 5

CFRD is a binary, time-varying CF-related diabetes indicator

t_{ik_i} is time of onset of CFRD in individual i

cPA is a binary, time-varying chronic pseudomonas aeruginosa indicator

t_{il_i} is time of onset of cPA in individual i

V_i is a random intercept with $V_i \sim N(0,\sigma_V^2)$

Z_{ij} is measurement error with $Z_{ij} \sim N(0,\sigma_Z^2)$

$W_i(t_{ij})$ differs between the UK and Danish models; in the UK model $W_i(t_{ij})=W_i t_{ij}$ is a random slope term with $W_i \sim N(0,\sigma_{W\_UK}^2)$, in Denmark $W_i(t)$ is a stationary Gaussian process with $W_i(t_{ij}) \sim N(0,\sigma_{W\_D}^2)$ and exponential correlation function $\rho(u)=Corr(W_i(t_{ij}), W_i(t_{ij}-u))=\exp(-|u|/\phi)$; reasons for the difference are given in the main text.

Model of seasonal effects using a sine function

We assume smooth changes of lung function between seasons and model this using a sine function with period one year,

$$\gamma_0 \sin(2\pi d/T) + \gamma_1 \cos(2\pi d/T)$$

where d is the day of the year on which the measurement was taken and T=365.25 days. The amplitude of this function is $\alpha = \sqrt{\gamma_0{}^2 + \gamma_1{}^2}$ and the horizontal shift, which determines the days of the year on which the function reaches its maximum and minimum values, is given by

$\Theta = \frac{T}{2\pi} \arctan(\gamma_1/\gamma_0)$ (see Figure S1).

Inclusion in the model for lung function is as follows:

$$y_{ij} = x'\beta_1 + x't_{ij}\,\beta_2 + CFRD(t_{ij})(t_{ij} - t_{ik_i})\beta_3 + cPA(t_{ij})(t_{ij} - t_{il_i})\beta_4$$

$$+\gamma_0 \sin(2\pi d_{ij}/T) + \gamma_1 \cos(2\pi d_{ij}/T) + V_i + W_i(t_{ij}) + Z_{ij}$$

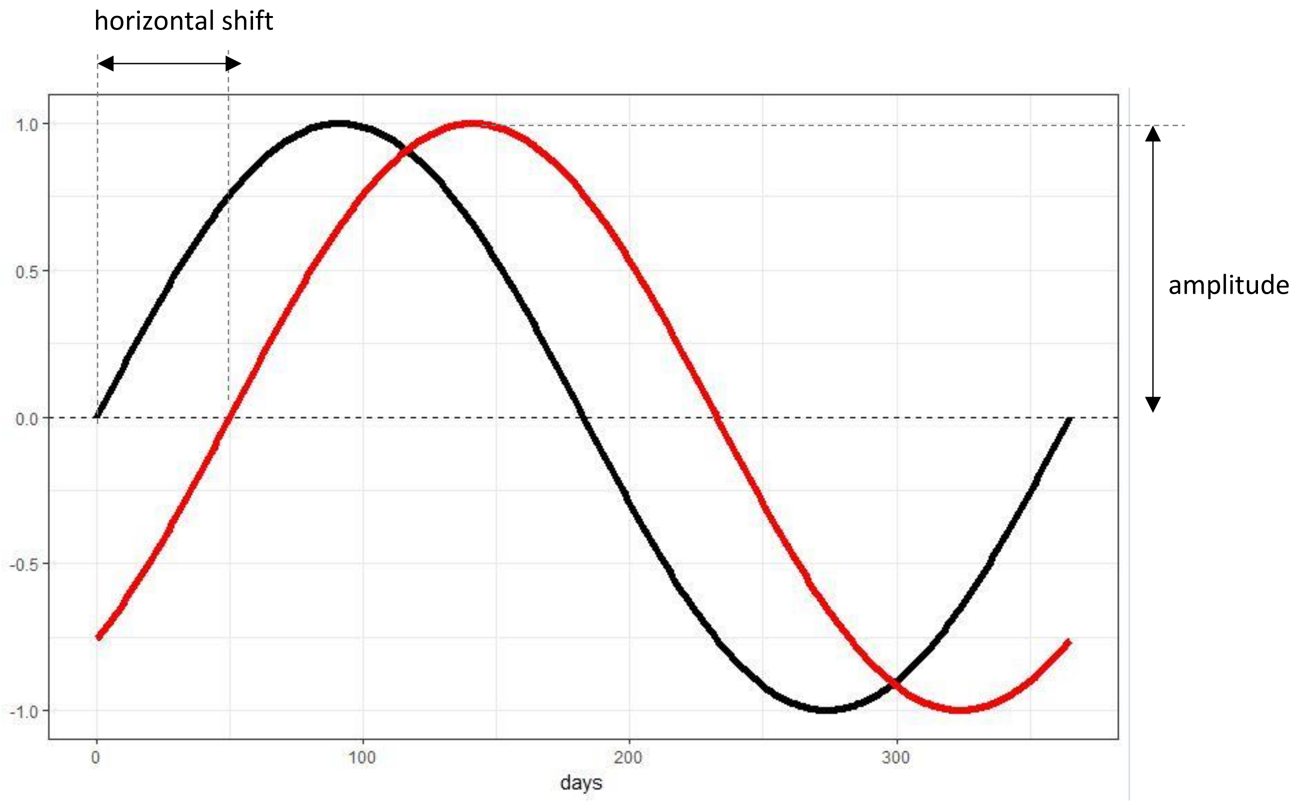

Figure S1: Sine waves with an amplitude of 1 and a period of 365.25 days. The red curve has a horizontal shift of 50 days in comparison to the black curve.

The confidence region for the amplitude α and for the horizontal shift θ are estimated by first finding the 95% confidence ellipse of (γ_0, γ_1) using a bivariate normal approximation. The joint confidence region for α and θ, is then the region of points ($\alpha=\sqrt{\gamma_0^{*2} + \gamma_1^{*2}}$, $\theta=\frac{T}{2\pi}\arctan(\gamma_1^*/\gamma_0^*)$) for all (γ_0^*, γ_1^*) in the 95% confidence ellipse for (γ_0, γ_1). This is given in Figure 1 in the main paper. It can be seen that the conditional 95% confidence interval for α varies for different values of θ and vice versa.

The unconditional 95% confidence intervals for α and θ are given by the ranges of their values over all values for (γ_0, γ_1) in the 95% confidence ellipse and are stated as confidence intervals in the main paper.

Results

Demographics

Table S1: Demographics of study population and for all individuals in the UK who were born between 1969 and 2010 and had at least one lung function measurement over the age of 5 (Eligible population).

	Study population	Eligible population
n	7586	9667

sex = male (%)	3977 (52.4)	5066 (52.4)
ethnicity = white (%)	7320 (96.5)	9348 (96.7)
Diagnosis by newborn screening= no (%)	6495 (85.6)	8246 (85.3)
F508del class (%)		
Heterozygous	2796 (36.9)	3408 (35.3)
Homozygous	4153 (54.7)	5011 (51.8)
Other	637 (8.4)	798 (8.3)
Missing		450 (4.7)
Mean IMD z-score (sd)	-0.01 (0.99)	0.00 (0.99)
CFRD during study period (%)		
no	5092 (67.1))	5768 (59.7)
yes	2494 (32.9)	3130 (32.4)
NA		769 (8.0)
Pancreatic insufficient (%)		
no	741 (9.8)	1010 (10.4)
yes	6845 (90.2)	8597 (88.9)
NA		60 (0.6)
Chronic PA during study period=yes (%)	4586 (60.5)	5711 (59.1)
Mean age at diagnosis (sd)	2.85 (6.53)	2.94 (6.66)

Parameters estimates for covariate effects

Table S2:Parameter estimates and 95% confidence intervals for the models fitted to the Danish data rounded to two digits

	Model with categorical season variable			Model with sine wave		
**Covariates affecting the intercept**	LCL	Estimate	UCL	LCL	Estimate	UCL
Intercept	76.48	100.15	123.81	76.55	100.21	123.88
age	-13.15	-4.41	4.34	-13.16	-4.41	4.33
Cohort 1968	-47.58	-29.78	-11.99	-47.57	-29.77	-11.97
Cohort 1978	-31.54	-13.81	3.92	-31.53	-13.8	3.93
Cohort 1988	-22.14	-4.59	12.96	-22.13	-4.58	12.97
Cohort 1998	-19.6	-1.97	15.66	-19.59	-1.96	15.67
Pancreatic insufficient	-19.99	-3.65	12.69	-20	-3.65	12.69
F508 class other	-25.12	4.52	34.16	-25.12	4.52	34.16
F508 class heterozygous	-16.23	-1.48	13.28	-16.24	-1.48	13.27
Sex=male	-1.32	2.92	7.16	-1.32	2.92	7.16
Age at diagnosis	-0.04	0.02	0.08	-0.04	0.02	0.08
Season summer	-0.07	0.1	0.28			
season spring	-0.15	0.01	0.18			
Season autumn	-0.03	0.13	0.3			
sine				-0.17	-0.09	0
cosine				-0.15	-0.06	0.03
**Covariates affecting the slope**						
Cohort 1968	-4.58	4.12	12.82	-4.57	4.13	12.83
Cohort 1978	-5.46	3.24	11.94	-5.45	3.25	11.95
Cohort 1988	-5.79	2.91	11.61	-5.78	2.92	11.62
Cohort 1998	-5.04	3.67	12.39	-5.03	3.68	12.39

Pancreatic insufficiency	-0.82	0.1	1.02	-0.82	0.1	1.02
F508 class other	-0.43	1.18	2.8	-0.43	1.18	2.8
F508 class heterozygous	0.39	1.2	2	0.39	1.2	2
Sex=male	-0.2	0.02	0.24	-0.2	0.02	0.24
Age at diagnosis	-0.01	0	0.01	-0.01	0	0.01
CFRD	-0.63	-0.32	-0.02	-0.62	-0.32	-0.02
Chronic PA	-0.9	-0.66	-0.41	-0.9	-0.66	-0.41

Table S3: Parameter estimates and 95% confidence intervals for the models fitted to the UK data rounded to two digits

	Model with categorical season variable			Model with sine wave		
**Covariates affecting the intercept**	LCL	Estimate	UCL	LCL	Estimate	UCL
Intercept	89.85	93.13	96.42	90.03	93.31	96.6
age	0.39	1.47	2.55	0.39	1.47	2.56
Cohort 1968	-16.24	-12.64	-9.03	-16.22	-12.62	-9.02
Cohort 1978	-8.38	-5.53	-2.67	-8.36	-5.51	-2.66
Cohort 1988	-3.07	-0.44	2.19	-3.05	-0.43	2.2
cohort 1998	-3.22	-0.64	1.94	-3.2	-0.63	1.95
Sex=male	-0.12	0.88	1.89	-0.12	0.88	1.89
F508 class heterozygous	-2.17	-1.06	0.06	-2.17	-1.05	0.06
F508 class other	-3.72	-1.62	0.48	-3.72	-1.62	0.48
IMD z-score	-1.34	-0.83	-0.32	-1.33	-0.83	-0.32
Ethnicity Black	-3.24	5.11	13.45	-3.27	5.08	13.43
Ethnicity SE Asian	-17	13.56	44.13	-16.99	13.58	44.14
Ethnicity Other/Mixed	-6.04	-2.94	0.16	-6.04	-2.93	0.17
Diagnosis by NBS	-1.89	-0.37	1.15	-1.89	-0.37	1.15
Pancreatic insufficiency	-5.95	-3.83	-1.7	-5.96	-3.83	-1.71
Age at diagnosis	0	0.14	0.27	0	0.14	0.27
Season summer	-0.04	0.21	0.45			
Season spring	0.1	0.34	0.58			
Season autumn	-0.06	0.17	0.39			
sine				-0.06	0.06	0.18
cosine				-0.25	-0.13	-0.01
**Covariates affecting the slope**						
Cohort 1968	-3.24	-2.16	-1.08	-3.24	-2.16	-1.08
Cohort 1978	-3.53	-2.46	-1.38	-3.53	-2.46	-1.39
Cohort 1988	-3.79	-2.72	-1.65	-3.79	-2.72	-1.65
Cohort 1998	-3.37	-2.3	-1.22	-3.37	-2.3	-1.23
Sex=male	0.05	0.13	0.21	0.05	0.13	0.21
F508 class heterozygous	0.1	0.19	0.27	0.1	0.19	0.27
F508class other	0.1	0.26	0.42	0.1	0.26	0.42
IMD z-score	-0.06	-0.02	0.02	-0.06	-0.02	0.02
Ethnicity Black	-1.37	-0.63	0.11	-1.37	-0.63	0.11
Ethnicity SE Asian	-2.91	-0.73	1.44	-2.92	-0.74	1.44
Ethnicity Other/Mixed	-0.45	-0.19	0.06	-0.45	-0.19	0.06
Diagnosis by NBS	0.12	0.25	0.39	0.12	0.25	0.39

Pancreatic insufficiency	-0.61	-0.45	-0.28	-0.61	-0.45	-0.28
Age at diagnosis	0	0.01	0.02	0	0.01	0.02
CFRD	-0.4	-0.31	-0.22	-0.4	-0.31	-0.22
Chronic PA	-0.24	-0.17	-0.09	-0.24	-0.17	-0.09

Estimates for Variance-Covariance parameters

Table S4: Estimates and 95% confidence intervals for the Variance-Covariance parameters in the models fitted to the Danish data rounded to two digits.

		Model with categorical season variable			Model with sine wave		
		LCL	Estimate	UCL	LCL	Estimate	UCL
Standard deviation of random effects on the intercept		11.85	13.96	16.44	11.92	13.96	16.34
Standard deviation of the remaining variation between measurements		16.28	17.72	19.3	16.36	17.72	19.18
Correlation structure	range	6.79	8.45	10.51	6.89	8.45	10.35
	Nugget	0.12	0.14	0.17	0.12	0.14	0.16

The total variance between observations is estimated to be 509; the between individual variance is 195; within individual variation is 270 and the error variance is 44. Figure S2 below shows the empirical variogram (black) and the theoretical variogram based on the above estimates (red).

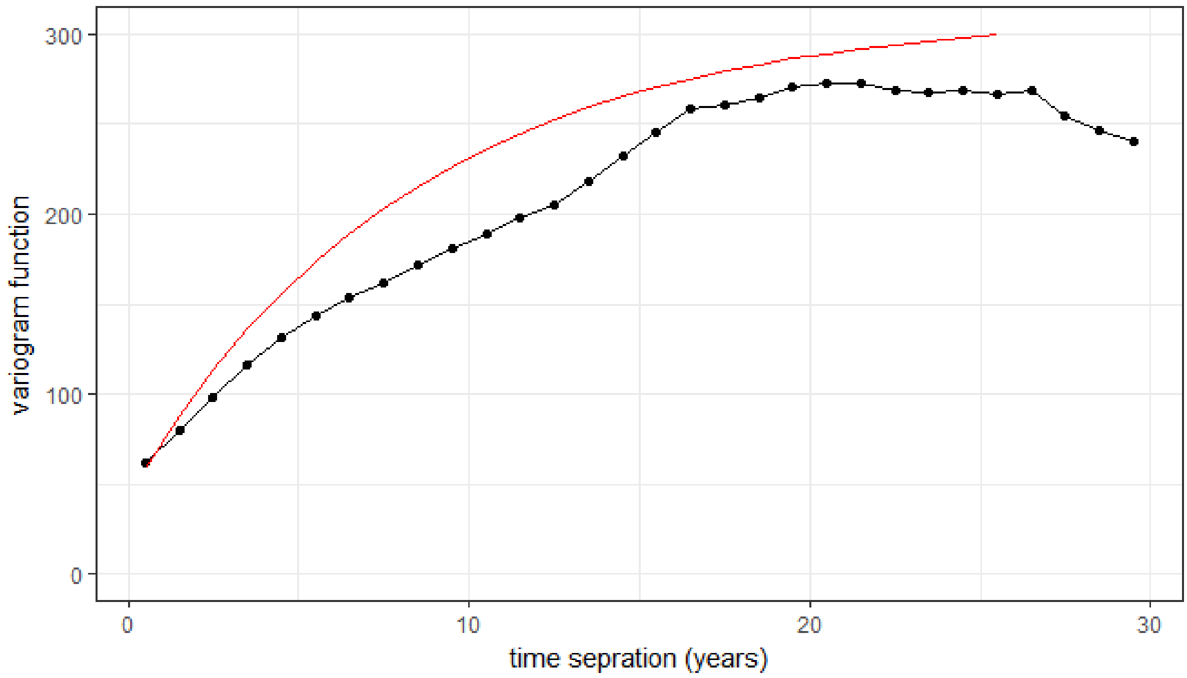

Figure S2: Empirical variogram (black) and the theoretical variogram based on the parameter estimates (red).

Table S5: Estimates and 95% confidence intervals for the Variance-Covariance parameters in the models fitted to the UK data rounded to two digits.

	Model with categorical season variable			Model with sine wave		
	LCL	Estimate	UCL	LCL	Estimate	UCL
Standard deviation of random effects on the intercept	18.65	19.07	19.5	18.65	19.07	19.5
Standard deviation of random effect on slope	1.36	1.4	1.43	1.36	1.39	1.43
Standard deviation of random error	8.44	8.49	8.54	8.44	8.49	8.54

Robustness test

The parameter estimates for the UK CF population born after 1991 are given in the tables below. Table S6 summarises the seasonal effect and Table S7 gives all the model parameters.

Table S6: Estimates and 95% confidence intervals for the amplitude and horizontal shift from model fit to the UK CF population born after 1991

**Parameters**	**Estimate (95% CI)**
**Amplitude of sine wave**	0.08 (0 to 0.34)
**Horizontal shift of sine wave**	19.02 (-182.62 to 182.29)

Table S7: Parameter estimates and 95% confidence intervals for the models fitted to the UK data from individuals born after 1991 rounded to two digits

	Model with categorical season variable			Model with sine wave		
**Covariates affecting the slope**	LCL	Estimate	UCL	LCL	Estimate	UCL
Intercept	89.54	92.69	95.83	89.54	92.68	95.81
age	0.17	1.33	2.49	0.18	1.34	2.5
Cohort 1988	-2.56	-0.2	2.15	-2.56	-0.21	2.15
cohort 1998	-2.59	-0.33	1.92	-2.59	-0.34	1.92
Sex=male	0.82	1.88	2.93	0.82	1.88	2.93
F508 class heterozygous	-2.05	-0.87	0.31	-2.05	-0.87	0.31
F508 class other	-2.04	0.17	2.37	-2.04	0.16	2.37
IMD z-score	-1.93	-1.4	-0.87	-1.93	-1.4	-0.87
Ethnicity Black	-1.05	6.7	14.44	-1.04	6.7	14.45
Ethnicity SE Asian	-30.22	1.65	33.51	-30.21	1.66	33.53
Ethnicity Other/Mixed	-6.72	-3.67	-0.62	-6.71	-3.66	-0.61
Diagnosis by NBS	-1.29	0.13	1.55	-1.29	0.13	1.55

Pancreatic insufficient	-6.36	-4.21	-2.05	-6.36	-4.21	-2.05
Age at diagnosis	-0.25	0.02	0.29	-0.25	0.02	0.29
Season summer	-0.48	-0.08	0.32			
Season spring	-0.35	0.04	0.43			
season autumn	-0.39	-0.02	0.34			
sine				-0.12	0.08	0.28
cosine				-0.17	0.03	0.23
**Covariates affecting the slope**						
Cohort 1988	-3.6	-2.48	-1.35	-3.6	-2.48	-1.35
cohort 1998	-3.24	-2.11	-0.98	-3.24	-2.11	-0.98
Sex=male	0	0.13	0.25	0	0.13	0.25
F508 class heterozygous	0.06	0.2	0.34	0.06	0.2	0.34
F508 class other	-0.18	0.09	0.36	-0.18	0.09	0.36
IMD z-score	-0.07	-0.01	0.05	-0.07	-0.01	0.05
Ethnicity Black	-1.72	-0.79	0.15	-1.72	-0.79	0.15
Ethnicity SE Asian	-4.54	-1.19	2.15	-4.54	-1.2	2.15
Ethnicity Other/Mixed	-0.63	-0.24	0.16	-0.63	-0.24	0.16
Diagnosis by NBS	0.12	0.31	0.5	0.12	0.31	0.5
Pancreatic insufficient	-0.72	-0.44	-0.16	-0.72	-0.44	-0.16
Age at diagnosis	-0.01	0.02	0.04	-0.01	0.02	0.04
CFRD	-0.78	-0.58	-0.37	-0.78	-0.58	-0.37
Chronic PA	-0.59	-0.46	-0.33	-0.59	-0.46	-0.33

Additional analysis

The parameter estimates for the models including an interaction between the sine wave and age group are given in the tables below. Table S8 summarises the seasonal effects and Table S9 contains all the parameter estimates for the models.

Likelihood ratio tests comparing the models with and without the age group interaction were not statistically significant (p-values of 0.65 and 0.13 in Denmark and the UK, respectively)

There was no significant difference in the seasonal patterns in lung function between children and adults in the UK or Denmark. The amplitudes were of no statistical or clinical significance in either country. In Denmark lung function was estimated to peak at the end of August in both children and adults with the confidence interval covering the entire year. In the UK lung function was estimated to peak on the 11th April in children and the 6th July in adults. The confidence interval for the peak of lung function in children covered the entire years whereas the confidence interval for the peak in adults ran from the 2nd April to 10th September.

Table S8: Estimates and 95% confidence intervals for the seasonal amplitude and horizontal shift variables from models which include an interaction terms between the sine wave and age group (<18 and ≥18)

Parameters	Estimate (95% CI) Denmark		Estimate (95% CI) UK	
	children	adults	children	adults
**Amplitude of sine wave**	0.07 (0, 0.25)	0.15 (0, 0.33)	0.15 (0,0.42)	0.22 (0, 0.41)
**Horizontal shift of sine wave**	149.26 (-182, 182)	147.86 (-182, 182)	9.66 (-179.12, 181.43)	95.32 (0, 160.75)

Table S9: Parameter estimates and 95% confidence intervals for the models including an interaction between sine wave and age group (<18 and ≥18) rounded to two digits.

	Denmark			UK		
**Covariate affecting the intercept**	LCL	estimate	UCL	LCL	estimate	UCL
Intercept	76.55	100.22	123.88	90.06	93.34	96.63
age	-13.16	-4.42	4.33	0.39	1.47	2.55
Cohort 1968	-47.57	-29.77	-11.97	-16.24	-12.64	-9.03
Cohort 1978	-31.53	-13.8	3.93	-8.39	-5.54	-2.68
Cohort 1988	-22.13	-4.59	12.96	-3.07	-0.44	2.19
Cohort 1998	-19.59	-1.96	15.67	-3.23	-0.66	1.92
Pancreatic insufficient	-20	-3.66	12.69	-5.96	-3.84	-1.71
F508 class heterozygous	-16.24	-1.48	13.27	-2.17	-1.05	0.07
F508 class Other	-25.11	4.53	34.17	-3.73	-1.63	0.47
Sex=male	-1.32	2.92	7.16	-0.12	0.89	1.89
IMD z-score				-1.33	-0.83	-0.32
Ethnicity Black				-3.24	5.11	13.46
Ethnicity SE Asian				-17	13.57	44.14
Ethnicity Other/Mixed				-6.01	-2.91	0.19
Diagnosis by NBS				-1.89	-0.37	1.15
Age at diagnosis	-0.04	0.02	0.08	0	0.14	0.27
sine	-0.27	-0.13	0.01	-0.18	-0.02	0.15
cosine	-0.23	-0.09	0.05	-0.38	-0.22	-0.05
Sine:age group<18	-0.11	0.07	0.25	-0.08	0.16	0.4
Cosine:age group<18	-0.13	0.05	0.23	-0.04	0.19	0.43
**Covariates affecting the slope**						
Cohort 1968	-4.57	4.13	12.83	-3.23	-2.16	-1.08
Cohort 1978	-5.45	3.25	11.95	-3.53	-2.46	-1.38
Cohort 1988	-5.78	2.92	11.62	-3.79	-2.72	-1.65
Cohort 1998	-5.03	3.68	12.39	-3.37	-2.29	-1.22
Pancreatic Insufficiency	-0.82	0.1	1.02	-0.61	-0.45	-0.28
F508 class Heterozygous	0.39	1.2	2	0.1	0.19	0.27
F508 class Other	-0.43	1.18	2.8	0.1	0.26	0.42
Sex=male	-0.2	0.02	0.24	0.05	0.13	0.21
IMD z-score				-0.06	-0.02	0.02
Ethnicity Black				-1.37	-0.63	0.11
Ethnicity SE Asian				-2.91	-0.74	1.44
Ethnicity Other/Mixed				-0.45	-0.2	0.06
Diagnosis by NBS				0.12	0.25	0.39
Age at diagnosis	-0.01	0	0.01	0	0.01	0.02
CFRD	-0.62	-0.32	-0.02	-0.4	-0.31	-0.22
Chronic PA	-0.9	-0.66	-0.41	-0.24	-0.17	-0.09
